# Is TKA femoral implant stability improved by pressure applied cement? a comparison of 2 cementing techniques

**DOI:** 10.1186/s12891-023-06151-0

**Published:** 2023-01-21

**Authors:** Mareike Schonhoff, Nicholas A. Beckmann, Martin Schwarze, Marvin Eissler, J. Philippe Kretzer, Tobias Renkawitz, Sebastian Jaeger

**Affiliations:** 1 Laboratory of Biomechanics and Implant Research, Department of Orthopaedics, Heidelberg University Hospital, 69118 Heidelberg, Germany; 2 Department of Orthopaedics, Heidelberg University Hospital, 69118 Heidelberg, Germany; 3 Department of Diagnostic and Interventional Radiology, Heidelberg University Hospital, Heidelberg, Germany

**Keywords:** Total knee arthroplasty, Cementing technique, Pressurizer, Loosening, Relative motion

## Abstract

**Background:**

The majority of knee endoprostheses are cemented. In an earlier study the effects of different cementing techniques on cement penetration were evaluated using a Sawbone model. In this study we used a human cadaver model to study the effect of different cementing techniques on relative motion between the implant and the femoral shaft component under dynamic loading.

**Methods:**

Two different cementing techniques were tested in a group of 15 pairs of human fresh frozen legs. In one group a conventional cementation technique was used and, in another group, cementation was done using a pressurizing technique. Under dynamic loading that simulated real life conditions relative motion at the bone-implant interface were studied at 20 degrees and 50 degrees flexion.

**Results:**

In both scenarios, the relative motion anterior was significantly increased by pressure application. Distally, it was the same with higher loads. No significant difference could be measured posteriorly at 20°. At 50° flexion, however, pressurization reduced the posterior relative motion significantly at each load level.

**Conclusion:**

The use of the pressurizer does not improve the overall fixation compared to an adequate manual cement application. The change depends on the loading, flexion angle and varies in its proportion in between the interface zones.

## Background

Osteoarthritis is the most common joint disease worldwide, affecting 344 million people [1]. The knee is the most frequently involved joint and accounts for 50% of cases [2]. Total knee arthroplasty is one of the most successful interventions for restoring knee joint function and reducing pain after nonsurgical treatment options have been exhausted and the patient’s quality of life has been permanently impaired [3]. In 2020 there were 111,365 primary knee replacements and 13,767 revisions registered in the German Arthroplasty Registry [4]. Cementation remains the gold standard in knee arthroplasty [5], even though numerous publications have reported comparable survival and functional outcome for both cemented and cementless fixation methods [3, 6, 7]. This trend is also confirmed in reports from several prosthesis registries, in which cementation was used in 68% to 94% of their cases [4, 8, 9].

Cementing technique has been the subject of scientific research for many years because it affects the crucial interface between prosthesis and bone and is intended to enable long term survival [10]. There are many factors influencing good cementation results including the type of cement, viscosity, volume used, mixing procedures, temperature, humidity, jet lavage, timing, speed and force during impaction of the components and handling of the cement [11–14]. Although loosening of the femoral components accounts for only 4.6% of revisions [4], the continuously increasing number of implantations and revisions, in addition to earlier reports of significantly higher loosening rates of modern high-flex prostheses has justified detailed investigations of methods for optimizing prosthesis fixation [15–18]. Also, recent publications have reported a higher incidence of radiolucent lines accompanying a new prosthesis design when compared to its predecessor, although the clinical and biomechanical significance of this is currently unclear [19, 20].

Studies were able to indicate that applying cement to both the bone and implant prior to implantation is advantageous in TKA [13, 14, 21, 22]. Also, several authors have demonstrated that using a cement gun is advantageous [14, 23, 24]. In a publication from 2019, Schwarze et al. were able to demonstrate in a Sawbone® model of knee arthroplasty that cement application with a pressurizer creates a more homogeneous cement coating and adequate cement penetration [12], thereby confirming positive results from prior studies [23–25]. However, the used Sawbone® model only partially reflects the in-vivo scenario in reference to the cement-bone inferface. The aim of the current study was to further investigate the effects of pressurized application of cement to human femoral cadaver specimens, in particular the effects on implant stability and relative motion of the implant/bone interface during loading.

## Methods

### General

This study was performed in accordance with the Declaration of Helsinki and approved by the local Ethics Committee (Ethikkommission der Medizinischen Fakultät Heidelberg, reference S-351/2018). The tissue samples were obtained from Science Care (Phoenix, AZ, USA), which is accredited by the American Association of Tissue Banks. All donors and/or their legal guardian(s) provided informed consent prior to sample acquisition.

In 15 pairs of fresh frozen human legs the Attune total knee replacement system (DePuy Synthes, Warsaw, IN, USA) was implanted by a surgeon experienced in the surgical technique. Preservation of biomechanical properties prior to the experimental period was ensured by frozen storage [26]. In a randomized manner, two different cementation techniques (Groups A and B) were used for the implantation of the femoral component of the Attune system.

Group A consisted of specimens in which the articular surfaces of the femoral components and of the femoral condyles all had conventional cement application using a cement gun. Both surfaces were covered with cement, as this has been shown to provide the best results [13]. Also, the cement gun has been shown to provide superior cementation of the bone to finger packing [14, 23, 24].

**Fig. 1:**
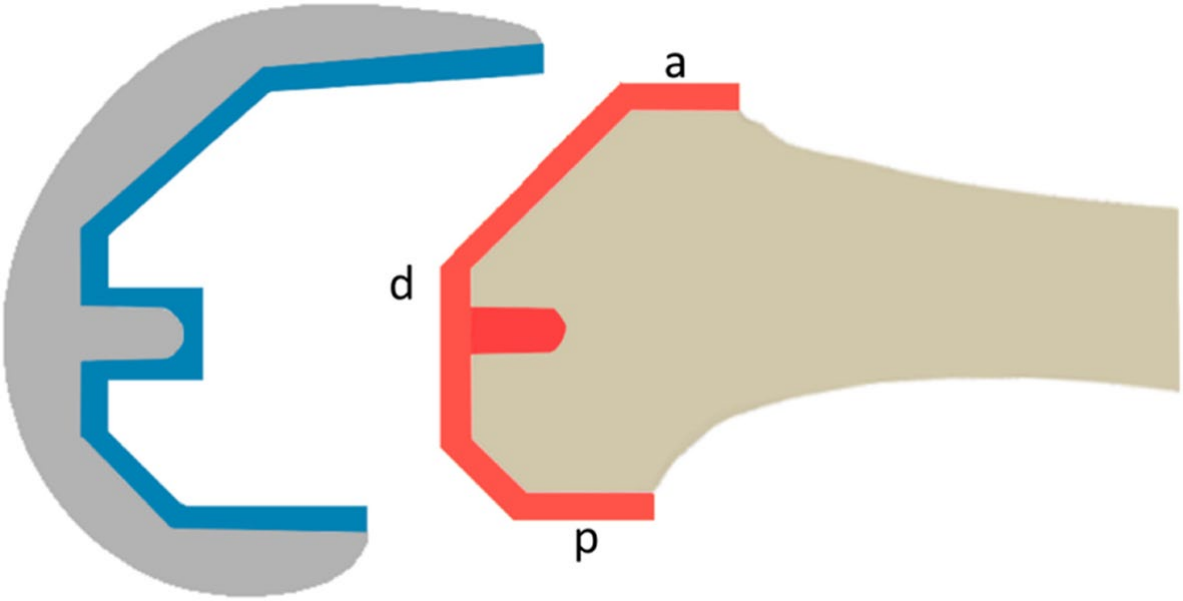
Femoral component and femur with red and blue colored areas on which cement has been applied. In Group A both areas were covered in the conventional manner; in Group B the red area indicates the femoral surface cemented using a no-touch technique implanted by use of a cement gun with a pressurizer. (**a**) is the anterior, (**d**) the distal and (**p**) the posterior regions

Group B had the cement applied to the distal femur with a pressurizing nozzle added to the cement gun. The cement was applied to the femoral component in the conventional manner as in group A. (Fig. 1).

More details on the cementation technique are provided below.

The right and left sides of the 15 leg pairs were randomly allocated to group A or B by means of a computer-generated list (Randlist 1.2; Datinf GmbH, Tübingen, Germany). The mean donor data showed a mean age of 68.3 ± 11.5 years, a mean height of 174.4 ± 10.9 cm, a mean weight of 75.1 ± 16.4 kg, and a mean body mass index of 24.6 ± 4.7 kg/m2.

The bone mineral density (BMD) was assessed for both groups to improve the comparability. Franck et al. showed a high correlation between standard dual-energy absorptiometry (DXA) at the hip and various locations such as the extremities [27]. Therefore, we measured bone mineral density using DXA with standard hip parameters (Hologic QDR-2000, Marlborough, Massachusetts, USA). For all 30 knee joints, native radiographs in anterior–posterior (a.p.) and lateral projections were obtained to exclude bone pathology that would preclude a knee prosthesis and to determine prosthesis size using TraumaCad software (Voyant Health, Ltd., Brainlab AG, Munich, Germany). The same prosthesis size was planned and implanted on the right and left side of each leg pair. The following prosthesis sizes were used: 5 × size 5, 3 × size 6, 3 × size 7, 4 × size 8. Postoperative radiographs were performed to verify the implantation result and to exclude intraoperative fractures.

### Cementing procedure

**Fig. 2:**
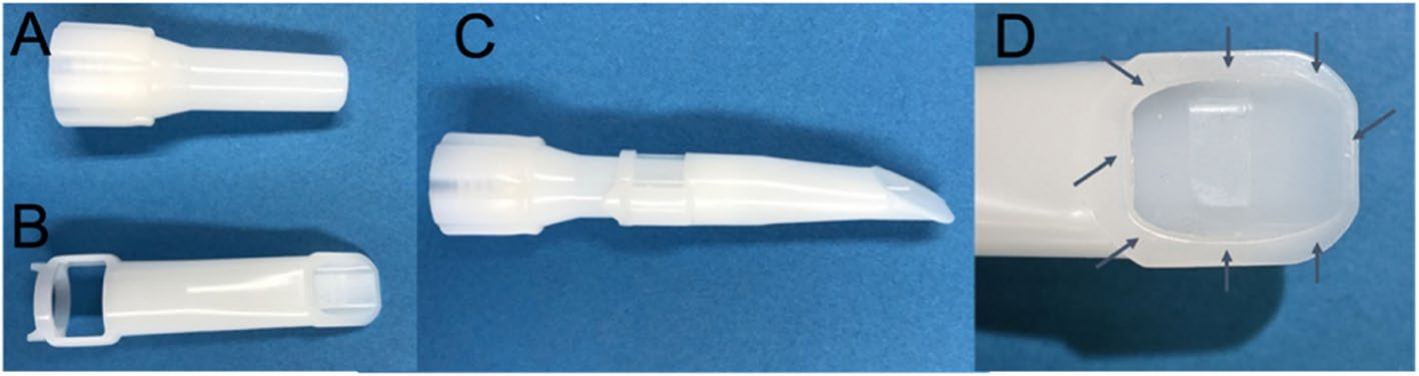
shows the standard nozzle short (**A**), the 23° pressurizer add-on (**B**), the attached 23° pressurizer on the standard nozzle short (**C**) and the contact surface between pressurizer and bone (**D**)

Prior to surgery, the human legs were thawed to room temperature. To standardize the experimental conditions and the surgical steps, all adjustments and resection measurements were documented and repeated on the contralateral side. Bone stock preparation and implantations were performed according to the prosthesis manufacturer's surgical instructions. The entire prosthesis was implanted and the femur and tibia were subsequently separated for testing. Prior to cementation, the cancellous bone was cleaned of lipid deposits, blood and bone debris using the OptiLavage system (Zimmer Biomet Holdings, Warsaw, Indiana, USA) and superficially dried with a compress until immediate cement application. The implantation of the femoral components for both Groups A and B was performed with a vacuum mixed high viscosity bone cement (Optipac 40 Refobacin Bone Cement R, Zimmer Biomet Holdings, Warsaw, Indiana, USA). The cement was applied early (in other words directly after the waiting phase) using cement timing for vacuum mixed cement at a room temperature of 21.2 ± 0.2 °C. We applied the bone cement to the non-articulating surface of the femoral components in Groups A and B 80 s after starting the mixing process. In the next step for Group A, the cement was applied to the prepared bone at 110 s using the above described cementing technique. In Group B, a cement gun with cement cartridge was also used, but a pressurizer nozzle was attached to the conventional nozzle to apply the cement to the bone in a no-touch technique (no manual manipulation of the cement after application) at 110 s (Fig. 2).

In group A, a homogeneous and uniform layer of PMMA cement was applied to the femoral bone stock with a cement gun (Optigun, Zimmer Biomet, Warsaw, Indiana, USA) medial and lateral from anterior to posterior for complete coverage. In addition, a homogeneous and uniform layer of cement was applied to the entire inner surface of the femoral knee component medially, laterally, and anteriorly transversely with the cement gun. On both the component and the bone, manual modelling with clean medical gloves was performed to ensure even coverage. In group B the cement gun was modified to deliver cement at an increased pressure by the attachment of a pressurizing nozzle with 23-degree angled tip. The cement was applied to the femoral surface under pressure, and the amount of cement was standardized to assess the influence of the cementing technique. An identical amount of cement was used in Groups A and B. The cement was applied to the femoral component in the same way in both groups [12].

The impaction of the femoral component was performed 140 s after start of mixing. The femoral component was impacted until the edges of the cement pockets were in contact with the distal bony resection surface. Excess cement was removed, and the trial liner insert was placed on the previously implanted tibial component. Subsequently, the leg was placed in extension position at 240 s after start of mixing, where the cement was allowed to harden.

### Load simulation and determination of relative motion

**Fig. 3:**
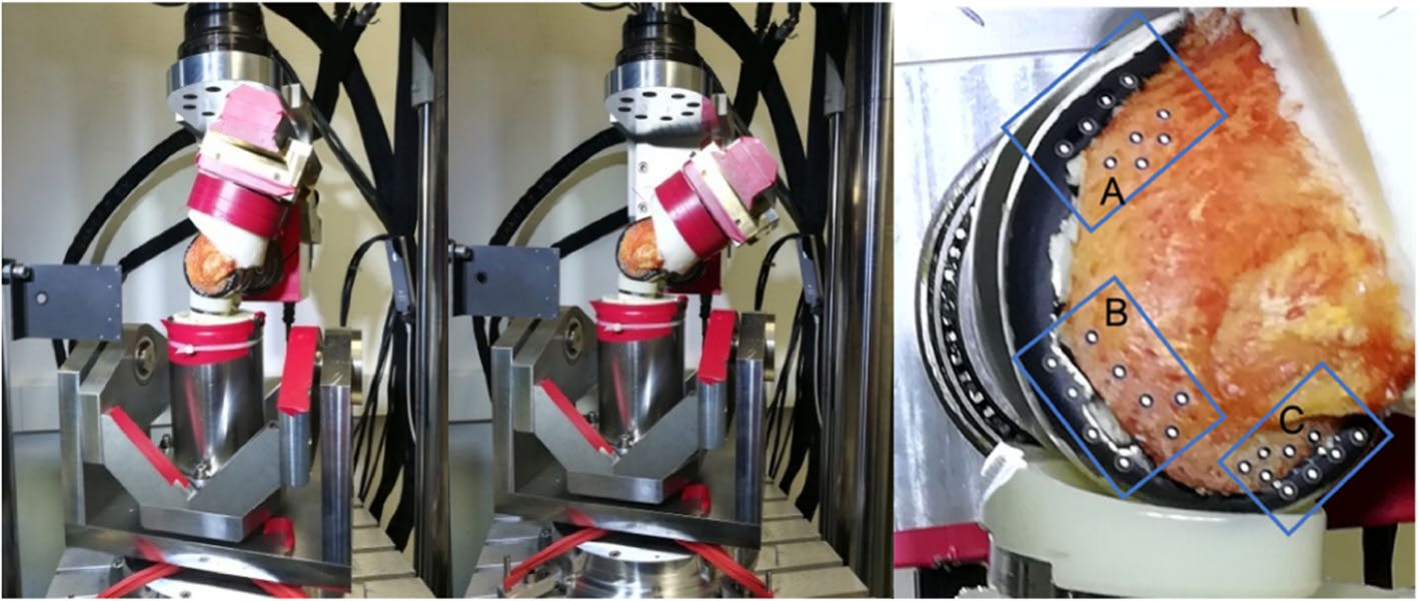
dynamic loading scenario of the distal femoral component in 20° (left) and 50° (center) of flexion articulating with the tibial liner in a servo-hydraulic testing machine. The casting resin in seen proximal to the femoral component. Correspondingly applied implant and bone markers (right) of the three analyzed zones (**A**: anterior, **B**: distal, **C**: posterior)

After the cementing procedure, the tibia and femur were separated, and the soft tissues removed. Afterwards, the specimens were cast in a mold using synthetic resin (Rencast FC 53, Huntsman Advanced Materials GmbH, Germany), in order to secure the specimens into the material testing machine. For the assessment of implant stability, an incremental dynamic load was applied at 1 Hz for the axial force with simultaneous extension-flexion between 20° and 50°, as had been done in a prior study [28]. The load maxima occurred at the time of extension and flexion, respectively [29]. A force representing daily stair climbing [30–32] was applied using a servo-hydraulic testing machine (MTS 858 Mini Bionix II, MTS Systems Corporation, Eden Prairie, USA) (Fig. 3). A preload of 200 N was applied before cyclic loading was started with the four load levels 1200 N, 1500 N, 1800 N, and 2100 N. The maximum load level corresponded to the force exerted on the knee of a person with a body weight of 75 kg during stair climbing [32]. The selected body weight for the load simulation corresponded to the average donor body weight. Optical markers were placed on the bone and the adjacent implanted component as shown in Fig. 3. The determination of the three-dimensional relative motion between the femoral component and bone was performed using an optical, camera-based system (PONTOS- GOM – Gesellschaft für Optische Messtechnik mbH, Braunschweig, Germany). Figure 3 shows the implant and bone markers (A: anterior, B: distal, C: posterior) of the three analyzed zones. The system is calibrated to a measurement volume of 250 × 200 mm^2^. The markers on the object to be measured were located in the center of this defined volume. Each of the markers were detected in greyscale by a stereo camera system, and a 3D point triangulation was done to calculate the 3D marker position and displacement vector in the defined coordinate system. The relative motion was calculated from the corresponding implant and bone markers. The two cameras of the stereo system operate each with a resolution of 2448 × 2050 pixels and a measuring accuracy of 1 µm according to the manufacturer's specifications [33]. However, the measuring accuracy of an optical measuring system depends strongly on the environmental conditions. Under laboratory conditions, we achieved a measuring accuracy of ± 4.9 µm for the test setup used. All measurements were done at the medial side. The calculated results of the resultant maximum relative motion were normalized to the right femur for maximum extension and flexion (20°, 50°).

### Statistical analysis

Prior to the start of the experimental study, a sample size calculation was performed using G*Power 3.1 (University of Kiel, Germany) [34] based on the reported data by Schwarze et al. [12]. The sample size differed (7, 9, 11, 16, 12 per group) depending on the cement penetration zone analyzed. The calculation of the sample size estimation with the largest number of cases per group was based on the following input parameters, tails: two, effect size d: 1.33, α err prob: 0.05 and power (1-β err prob): 0.95. This results in the output parameters sample size of 16 for each group. A sample size of 15 paired fresh frozen human legs was chosen for feasibility reasons. The data were evaluated descriptively using the arithmetic mean, standard deviation, minimum and maximum. Prior to analysis, the normal distribution of the data was evaluated using a Shapiro–Wilk-test and the homogeneity of variance was verified using the Levene-test. We conducted a two tailed t-test for independent samples to assess effects between both groups on the parameters BMD and relative motion within each load level, flexion angle and fixation zone. All data were analyzed using SPSS 25 (IBM, Armonk, NY, USA) with a significance level of *p* < 0.05.

## Results

15 fresh frozen pairs were acquired to carry out the experiments. During the radiographic evaluation, one pair was excluded due to a bone lesion and subsequent fracture during experimentation. We evaluated the remaining 14 pairs.

### Bone density

Testing the density differences between the two test groups using the Shapiro–Wilk test resulted in a *p*-value of 0.4. Thus, a normal distribution of the difference in bone density in both groups was confirmed. The following paired t-test showed no significant difference in bone density between the two groups (t(14) = -0.449, *p* = 0.66, d = 0.12).

### Relative motion

The determination of the resulting (XYZ) maximum relative motion between implant and bone was analyzed for all four load levels with a total of 4000 cycles. The points in time of the maximum extension-flexion values (20° and 50°) with simultaneous maximum axial load were analyzed. When analyzing the femoral components, the evaluation was also divided into anterior, distal and posterior regions.

### Femur 20º

The check for normal distribution with the Shapiro–Wilk test yielded (α = 0.05) normally distributed data. Therefore, a t-test was used for dependent samples. The test showed the following values for the load levels examined (Table. 1).

**Table 1:** Anterior, distal and posterior relative motion at 20° flexion of the femur depending on the load

		anterior		distal		posterior	
load level	*N* = 14	mean values ± SD (µm)	*p*-value	mean values ± SD (µm)	*p*-value	mean values ± SD (µm)	*p*-value
1200 N	Regular	10.0 ± .1.3	**0.011**	8.8 ± 1.2	0.158	11.8 ± 0.7	0.064
	Pressurizer	13.4 ± 2.9		8.1 ± 0.7		10.8 ± 0.7	
1500 N	Regular	14.5 ± 1.0	**0.001**	10.6 ± 0.4	**0.010**	13.3 ± 0.6	0.351
	Pressurizer	21.1 ± 2.4		11.9 ± 0.8		13.5 ± 0.6	
1800 N	Regular	18.4 ± 1.3	**< 0.001**	12.6 ± 0.9	**0.003**	15.7 ± 0.7	0.131
	Pressurizer	29.9 ± 2.8		14.6 ± 0.4		16.3 ± 1.2	
2100 N	Regular	24.8 ± 1.5	**< 0.001**	15.1 ± 0.8	**0.040**	20.3 ± 1.7	0.322
	Pressurizer	42.1 ± 3.4		16.3 ± 1.6		20.6 ± 1.4	

The max anterior relative motion at 20° flexion was at 2100 N for both groups: 53.7 µm for the group without a nozzle, and 130.7 µm for the group with nozzle

The max distal relative motion at 20° flexion was at 2100 N for both groups: 48.8 µm for the group without a nozzle, and 64.3 µm for the group with nozzle

The max posterior relative motion at 20° flexion was at 2100 N for both groups: 56.6 µm for the group without a nozzle, and 54.3 µm for the group with nozzle

### Femur 50º

The check for normal distribution with the Shapiro–Wilk test yielded.

(α = 0.05) normally distributed data. Therefore, a t-test was used for dependent samples. The test showed the following values for the load levels examined (Table. 2).

**Tab 2:** Anterior, distal and posterior relative motion at 50° flexion of the femur depending on the load

		anterior		distal		posterior	
load level	*N* = 14	mean values ± SD (µm)	*p*-value	mean values ± SD (µm)	*p*-value	mean values ± SD (µm)	*p*-value
1200 N	Regular	15.3 ± 1.0	**0.002**	7.6 ± 1.7	0.140	21.3 ± 1.1	**< 0.001**
	Pressurizer	22.2 ± 3.1		6.9 ± 1.5		16.5 ± 0.4	
1500 N	Regular	21.3 ± 1.2	**< 0.001**	10.2 ± 0.7	0.145	23.3 ± 0.4	**< 0.001**
	Pressurizer	32.2 ± 2.4		10.6 ± 1.1		19.7 ± 0.6	
1800 N	Regular	27.1 ± 1.8	**< 0.001**	12.6 ± 0.9	**0.028**	25.8 ± 0.2	**< 0.001**
	Pressurizer	39.8 ± 1.6		13.3 ± 0.6		21.8 ± 0.7	
2100 N	Regular	35.2 ± 1.7	**< 0.001**	15.0 ± 1.1	**0.013**	31.7 ± 0.9	**< 0.001**
	Pressurizer	46.8 ± 1.8		16.7 ± 0.8		23.2 ± 0.9	

The max anterior relative motion at 50° flexion was at 2100 N for both groups: 78.1 µm for the group without a nozzle, and 150.9 µm for the group with nozzle

The max distal relative motion at 50° flexion was at 2100 N for both groups: 55.6 µm for the group without a nozzle, and 81.3 µm for the group with nozzle

The max posterior relative motion at 50° flexion was at 2100 N for both groups: 79.1 µm for the group without a nozzle, and 70.4 µm for the group with nozzle

**Fig. 4:**
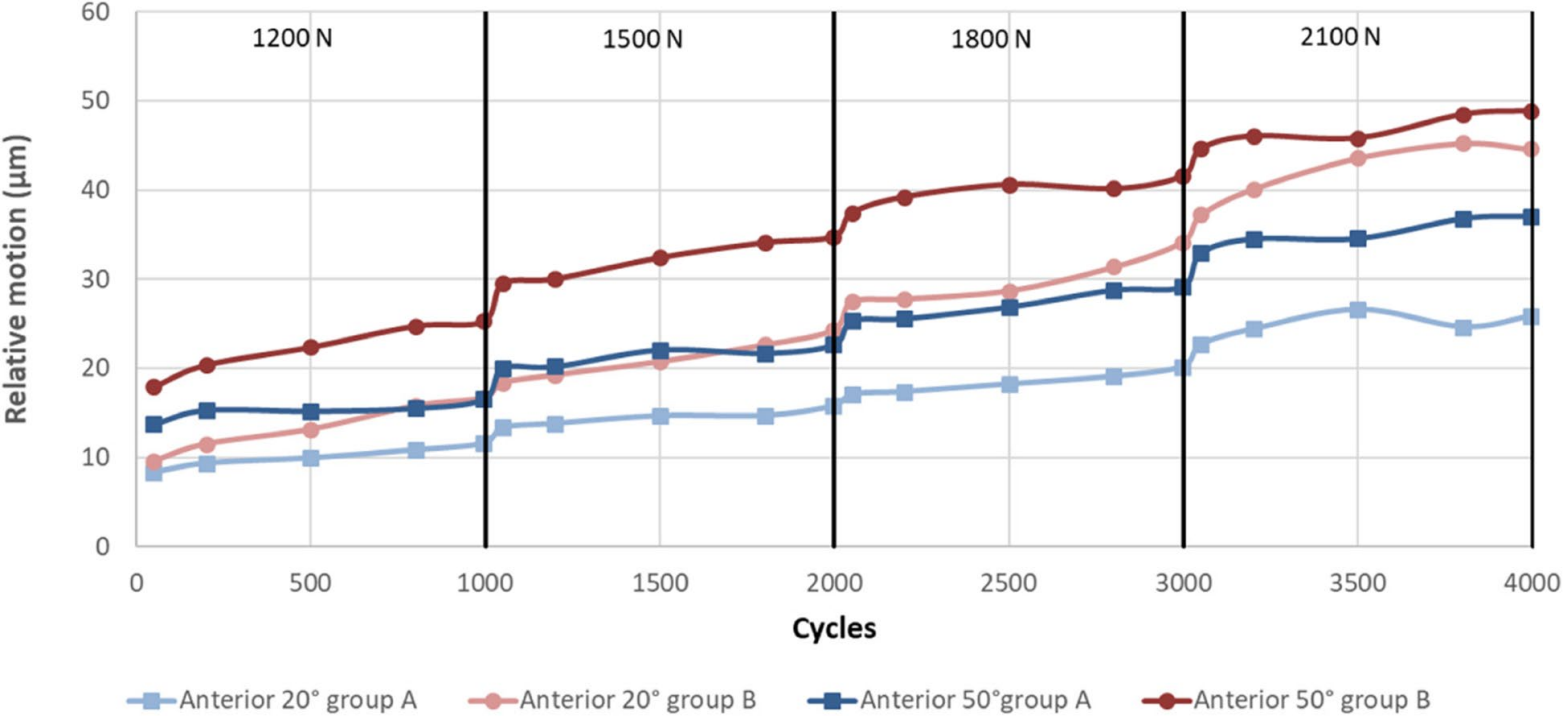
anterior relative motion as a function of the degree of flexion, group and incrementally increased load

**Fig. 5:**
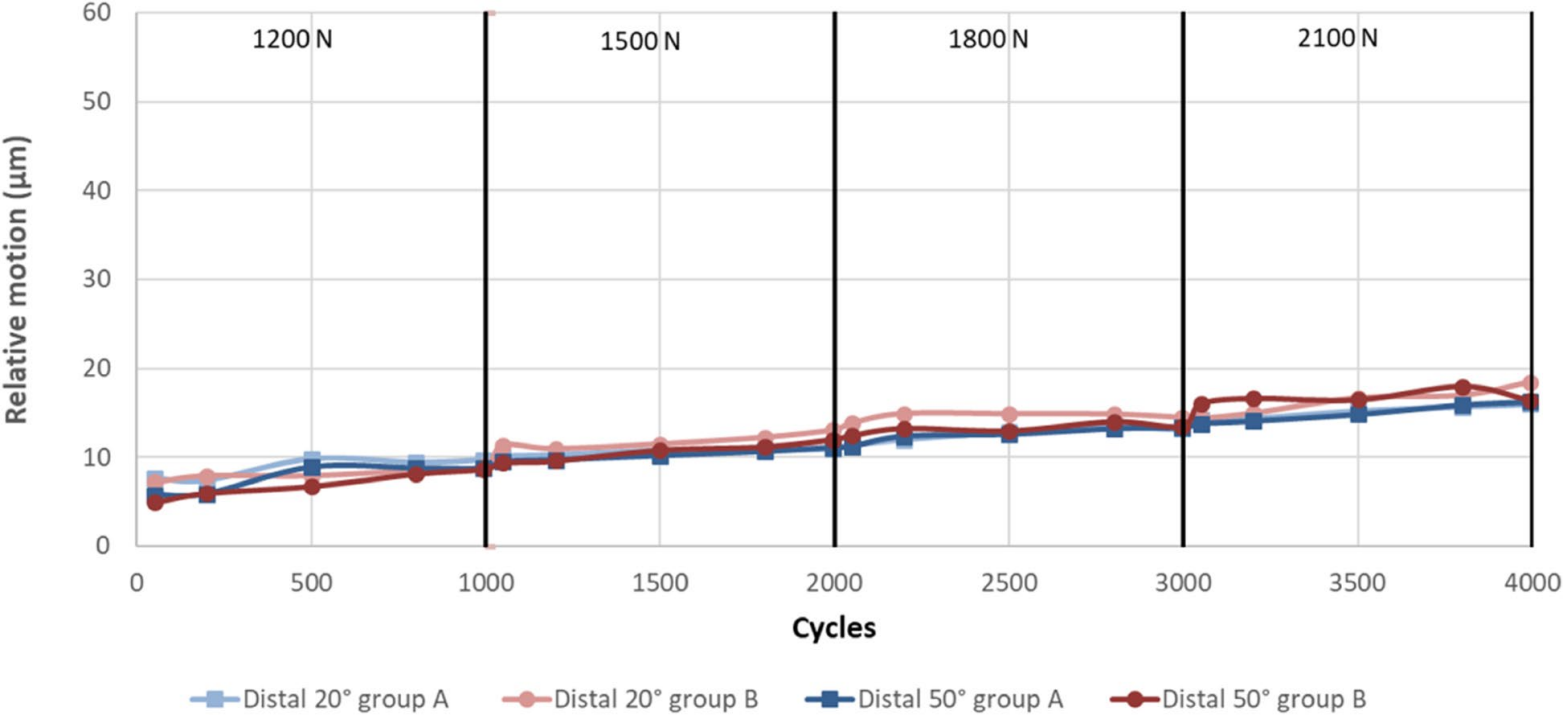
distal relative motion as a function of the degree of flexion, group and incrementally increased load

**Fig. 6:**
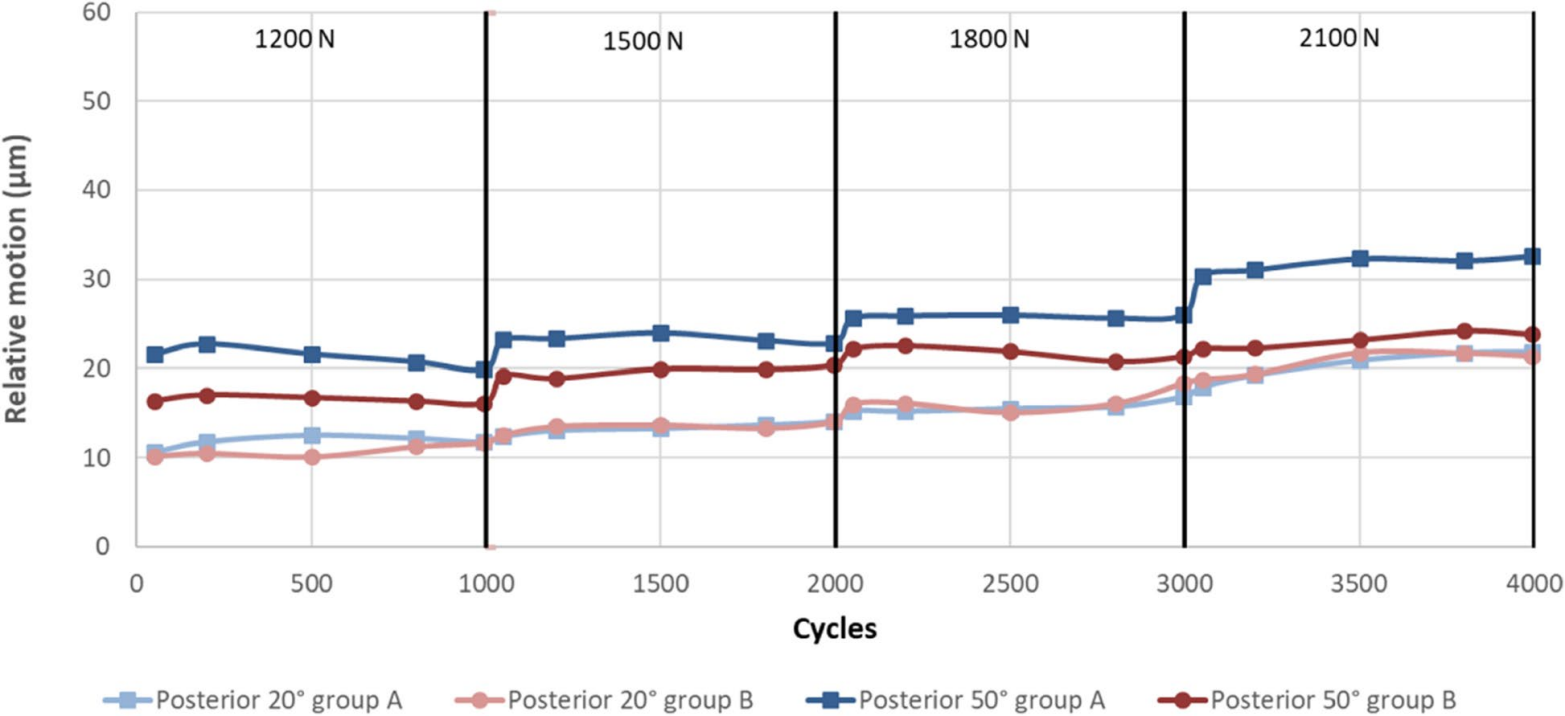
posterior relative motion as a function of the degree of flexion, group and incrementally increased load

Figures 4, 5 and 6 illustrate the anterior (Fig. 4), distal (Fig. 5) and posterior (Fig. 6) relative motion as a function of the degree of flexion (20˚ or 50˚), group (A or B) and incrementally increased load (1200 – 2100 N).

## Discussion

Although femoral component loosening is a relatively uncommon occurrence and constitutes only 4.6% of all total knee arthroplasty (TKA) revisions, it is a relevant complication based on the constantly increasing number of implantations and revisions [4, 12]. Studies have shown that cementation technique has a significant impact on femoral component loosening[14, 22]. Our results with human fresh-frozen specimens and cement pressurization show that at 20-degrees and 50-degrees of flexion and incrementally increasing load there is an increase in the relative motion between implant and bone for both cementation techniques. In addition, the increase in relative motion both anteriorly and distally with higher loads is significantly higher in the pressurized cementation group compared to non-pressurized cementation. In contrast, posteriorly at 50 degrees flexion, the relative motion between implant and bone is significantly reduced with cement pressurization compared to non-pressurized cementation. Our measured values of relative micromotion between the bone and the components fell between 7 and 46 µm, which are comparable to values recorded in prior studies [35–37].

There have been numerous prior in-vitro and in-vivo studies of the primary stability of hip and knee arthroplasties, using radiostereometric analyses (RSA) and optical measurements as utilized in our current study[37–42]. These have shown that many factors influence primary stability, including implant design, bone density, surgical technique and cement penetration [36, 43, 44].

Pressure application during cementation has been shown to be effective in increasing cement penetration [12, 23–25]. In 2019, Schwarze et al. published data on 3 different cementation techniques in a Sawbone® model [12] and showed that pressure application improved cement penetration in all zones of the Knee Society Scoring System (KSSS). Although the Sawbone® model had a bone structure similar to human cancellous bone [45], it differed from physiological bone in other properties and therefore may not reflect cement penetration in the clinical setting.

This study demonstrates that the cementation technique can significantly influence the degree of relative motion at the bone/femoral component interface under differing loading conditions. We found a significant reduction of relative motion posteriorly only at 50 degrees flexion at all load levels with pressurization, that we attribute to increased stability resulting from increased compression load during this degree of flexion. All other values of relative motion at both 20 and 50 degrees of flexion showed decreased relative motion in the non-pressurized samples, mostly of a significant degree. These latter findings were unexpected, and we have no good explanation. This may be related to differing elastic and plastic deformation in different areas of the bone implant construct. Less movement in the posterior region may result in more pronounced movement in the other regions. Further investigations would be helpful in this regard.

Improved distributions of bone cement using pressure application [12] can significantly affect force transmission at the cement-host bone interface [46]. In a finite element analysis, Schultze et al. described the influence of cement thickness and prosthesis positioning, with the highest von Mises stresses anteriorly [46]. Our results showed that using a pressurizer only achieved a significant reduction of the relative motion between the implant and bone in the posterior region. The very narrow posterior intercondylar space precludes accurately controlled manual cement application into cancellous bone during the surgical procedure. Our results suggest that pressurized clinical application might be helpful for improved cementation in the posterior region only. The clinical significance of our documented differences in relative motion is unclear.

We cannot determine any clear association between our results and the occurrence of radiolucent lines noted radiographically. Hoskins et al. reported the majority of radiolucent lines distally (34.5%) and anteriorly (6.9%) while Staats et al. described them as being predominantly posteriorly located (12%) [19, 20]. The authors therefore do not see any noticeable association with the results of the current study.

### Limitations

Although our experimental set-up mimicked the clinical situation as much as possible, the physiological effect of the surrounding soft tissues, differences in bone density and occurrence of bleeding could not be reproduced, limiting the extrapolation of our results to the clinical scenario.

Only two flexion angles were tested, unlike the physiological state which has a much greater range of motion.

The Attune knee replacement system was the only system tested and the results may vary with other systems.

Our incrementally increasing loads for 1000 cycles represent the immediate post-operative period only and does not reflect micromotion that could occur over the long-term postoperatively.

## Conclusions

Pressure application of bone cement changes the relative motion at the implant-bone interface in all areas. The change varied with the degree of loading and the joint flexion angle and differed in the anterior, distal and posterior bone/component interface zones (seen Table 2). Our results suggest that the use of the pressurizer did not improve the overall fixation compared to an adequate application using a cement gun, with the possible exception of the posterior zone. The posterior region was the only area that displayed a significant reduction of micromotion with pressurized cement application during flexion. Therefore, we suggest that application of cement with a pressurizer may be advantageous in this region only, where the narrow intercondylar space makes satisfactory manual application or use of a cement gun without a pressurizer difficult. An improved cementation technique may further decrease the component loosening seen clinically. Additional studies are suggested to investigate this further.

## Data Availability

The datasets used and/or analysed during the current study available from the corresponding author on reasonable request.
